# Safety, tolerability, pharmacokinetics, pharmacodynamics, and immunogenicity of STSA-1002, recombinant anti-human C5a fully human monoclonal antibody, in a randomized, first-in-human phase I study in healthy adults

**DOI:** 10.3389/fphar.2026.1838620

**Published:** 2026-06-18

**Authors:** Yingying Fang, Xiaoqian Wang, Lu Qi, Yuanxu Tong, Yali Wei, Zhong Li, Chen Liu, Ju Liu, Xiaoqiang Cheng, Xiaoyun Liu, Fang Liu, Yinjuan Li, Yan Li, Long Liu, Mingli Sun, Yu Wang, Xinghe Wang

**Affiliations:** 1 Department of Phase I Clinical Trial Center, Beijing Shijitan Hospital, Capital Medical University, Beijing, China; 2 Staidson Biopharmaceuticals (Beijing) Co., Ltd, Beijing, China

**Keywords:** ARDS, COVID-19, monoclonal antibody, STSA-1002, virus

## Abstract

STSA-1002 is a fully human monoclonal antibody targeting C5a, which plays a critical role in the pathogenesis of virus-induced acute respiratory distress syndrome ARDS (v-ARDS). We investigated safety, pharmacokinetics, pharmacodynamics, and anti-drug antibodies of increasing multiple intravenous doses of STSA-1002 in healthy adults. *In vitro* studies, STSA-1002 was a high-affinity, selective anti-human C5a monoclonal antibody blocking C5a-mediated reactive oxygen species (ROS) production. Building on the published preclinical results, a randomized, double-blind, placebo-controlled, dose-escalation study was done at a Phase I unit in China. A total of 60 adverse events (AEs) were reported. In the STSA-1002 group (n = 20), 47 AEs occurred in 18 participants (90.0%), corresponding to a mean of 2.6 AEs per participant. In the placebo group (n = 6), 13 AEs occurred in all 6 participants (100%), corresponding to a mean of 2.2 AEs per participant. Grade 2 or greater study drug-related adverse events included neutrophil count decreased, white blood cell count decreased, and aminotransferase increased. C_max_ and AUC_0-t_ increased in an approximately dose-proportional manner. After the first dose, the mean concentration of free C5a in all dose groups was suppressed to below the lower limit of quantification. This suppression was maintained in all health volunteers until D42 (5 mg/kg) and D56 (10 mg/kg and 20 mg/kg) after multiple doses. Besides, STSA-1002 could downregulate the levels of myeloperoxidase (MPO), neutrophil elastase (NE), and proteinase 3 (PR3). Overall, multiple infusions of STSA-1002 up to 20 mg/kg appear to be safe and well tolerated in healthy participants in China. The safety, PK, and PD data support the continued evaluation of STSA-1002 in larger Phase II studies.

**Clinical Trial Registration:** https://clinicaltrials.gov/, ID NCT05497635.

## Graphical Abstract

**Figure:**
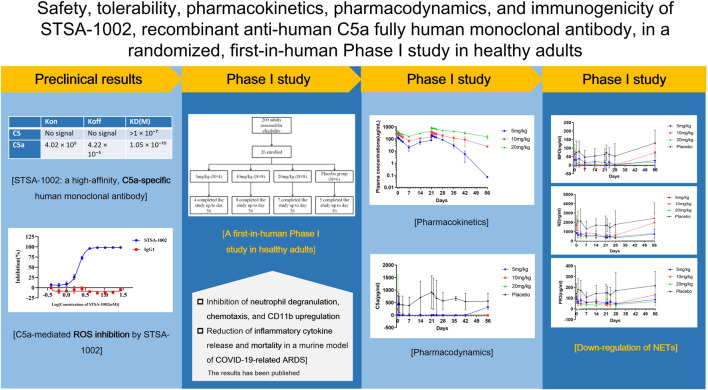

## Introduction

ARDS cases increased dramatically worldwide and carried a high mortality, especially among infections with highly pathogenic viruses, including influenza A viruses, Middle East respiratory syndrome coronavirus (MERS-CoV), severe acute respiratory syndrome (SARS) coronavirus 1 and SARS coronavirus 2 (COVID-19) (Wang et al., 2015; Grotberg et al., 2023). Unregulated complement activation is likely to play a crucial role in the pathogenesis of highly pathogenic virus-induced acute lung diseases despite the various epidemiologies (Meliopoulos et al., 2014). Overproduced anaphylatoxin (C5a) displays powerful biological activities in excessive complement activation (Wang et al., 2015).

C5a plays a critical role in the pathogenesis of virus-induced acute respiratory distress syndrome ARDS (v-ARDS). High concentrations of C5a and C5b-9 have been reported in patients with severe COVID-19, and one publication stressed the association of COVID-19 inflammation with activation of C5a (Carvelli et al., 2020). Growing evidence showed that increased levels of C5a were found in bronchoalveolar lavage fluid (BALF) and serum from patients infected with the fatal H1N1 pandemic virus (Garcia et al., 2013). Furthermore, several case series suggested C5a plays an important role in the outcome of ARDS induced by influenza virus and MERS-CoV infection, and targeting the complement system, especially C5a, may be an attractive therapeutic approach for combating v-ARDS (Song et al., 2018; Jiang et al., 2019; Zhang et al., 2021).

C5a binds to its receptor (C5aR1) on neutrophils, triggering their activation, degranulation, chemotaxis, and the formation of neutrophil extracellular traps (NETs)—a process known as NETosis. NETs are meshworks of decondensed chromatin fibers decorated with granular proteins such as myeloperoxidase (MPO), neutrophil elastase (NE), and proteinase 3 (PR3). While NETs normally serve to entrap and neutralize pathogens, uncontrolled NET release can promote inflammation, endothelial damage, and immunothrombosis, thereby exacerbating acute lung injury. Indeed, recent studies have mechanistically linked complement activation to the C5a NET axis in acute respiratory distress syndrome (ARDS). For instance, complement activation was shown to drive macrophage trafficking from the lungs to the blood in transfusion-related acute lung injury (TRALI) models, and NET formation was associated with C5a stimulation in the same setting (van der Velden et al., 2024). Moreover, in critically ill COVID-19 patients, elevated plasma MPO DNA complexes—a marker of NETs—were detected in lung autopsies of patients who required intubation and subsequently died, indicating that NETs contribute to immunothrombosis in COVID-19 ARDS (Middleton et al., 2020). In line with these observations, blocking the C5a NET axis with carboxypeptidase B (which inactivates C5a) was shown to reduce NET formation in neutrophils from COVID-19 patients (Zhang et al., 2021). Collectively, these findings highlight the C5a NET axis as a novel pathogenic mechanism and a promising therapeutic target in virus-induced ARDS.

STSA-1002 is a fully human IgG1 monoclonal antibody that specifically binds to human C5a with high affinity, which only blocks C5a effects but does not affect the formation of C5b-9 membrane attack complex. The preclinical profile of STSA-1002 has been previously characterized in our published studies. Specifically, we have reported that STSA-1002 effectively inhibited C5a-induced neutrophil degranulation, chemotaxis, and CD11b expression *in vitro* (Fang et al., 2025). *In vivo*, single intravenous administration of STSA-1002 (1, 3, and 10 mg/kg) tended to reduce mortality in a COVID-19-related LPS-induced ARDS mouse model with a significant decrease of inflammatory factors, including interleukin-6 (IL-6), tumor necrosis factor α (TNF-α), granulocyte-macrophage colony-stimulating factor (GM-CSF), and monocyte chemotactic protein-1 (MCP-1) (Yingying Fang and Wang, 2024; Fang et al., 2025). These previously published data established the preclinical rationale for the present first-in-human Phase I study. In addition, anti-C5a antibody (Vilobelimab) was reported to decrease the mortality of invasive mechanically ventilated patients (PaO_2_/FiO_2_ ratio of 60–200 mmHg) with COVID-19 in the PANAMO trial (Vlaar et al., 2022). The objectives of the current study were to characterize new results on the safety and tolerability of multiple doses of STSA-1002 in healthy adults and to perform a new assessment of the pharmacokinetics (PK), pharmacodynamics (PD), and anti-drug antibodies (ADA) of STSA-1002.

## Materials and methods

### Characterization of STSA-1002 binding affinity

To characterize the binding properties of STSA-1002, we performed surface plasmon resonance (SPR) analysis using a Biacore T200 system (GE Healthcare). The antibody was covalently immobilized onto a CM5 sensor chip, and its interaction with human C5a and C5 was evaluated by injecting a concentration series (0–10 nM). Real-time binding responses were monitored, and the association (k_on_) and dissociation (k_o_ff) rate constants were derived from the resulting sensorgrams. The equilibrium dissociation constant (K_D), a measure of binding affinity, was subsequently calculated.

### ROS release assay

C5a can stimulate neutrophils to release reactive oxygen species (ROS), promoting neutrophils to participate in a wide range of inflammatory reactions. Based on this biological activity mechanism, induced neutrophils were used to detect the blocking effect of anti-C5a antibodies. The cell line HL-60 is a human promyelocytic leukemia cell line. In brief, 1 mM di-butyryl cAMP sodium salt (Sigma, D0260) was used to induce HL-60 differentiation for 48 h; the cells decreased, became spindle-shaped, and differentiated toward neutrophils. A mixed solution of a series of concentrations of STSA-1002 and C5a (5 nM) was used to treat the differentiated cell. DCFH-DA fluorescence probe (Sigma, D6883) was added after 30 min. After incubation, fluorescence intensity was measured at an excitation wavelength of 480 nm and an emission wavelength of 525 nm. The data was normalized to calculate the inhibitory activity of the antibody, with only C5a stimulation and free of C5a stimulation as 0% and 100% inhibition, respectively.

### Study design

This was a first-in-human, randomized, double-blind, placebo-controlled, dose-escalation Phase Ib study (ClinicalTrials.govidentifier: NCT05986877) designed to evaluate the safety, tolerability, pharmacokinetics, and pharmacodynamics of multiple intravenous doses of STSA-1002 in healthy volunteers. Participants were enrolled into three sequential dose cohorts (5, 10, and 20 mg/kg) and randomized to receive either STSA-1002 or placebo in a 2:1 ratio for the 5 mg/kg cohort and a 4:1 ratio for the 10 and 20 mg/kg cohorts. The study consisted of a 4-week treatment period during which subjects received a single intravenous infusion once weekly, followed by a follow-up period extending to Day 56 post-last dose. The study protocol was approved by the institutional review board and independent ethics committee at each participating site. All subjects provided written informed consent before enrollment, and the study was conducted in accordance with the principles of the Declaration of Helsinki and Good Clinical Practice guidelines.

### Randomization and masking

Randomization was done with a computer-generated randomization schedule and in accordance with the randomization plan prepared and maintained by CIMS-Central Randomization System (Chengdu CIMS Medtech Co., Ltd., China). To maintain the masking of the study, the randomization plan was available only to the unmasked pharmacist. Treatment allocation was double-blinded, and STSA-1002 and placebo were identical in appearance and volume for each participant in each cohort.

### Patient population

Key inclusion criteria included: male or female patients aged ≥18 years and ≤45 years, with a body-mass index of 19.0 kg/m^2^-26.0 kg/m^2^. All participants were required to have normal laboratory parameters at enrollment, and could not have any chronic medical problem that required daily oral medications, a positive immunoglobulin E factor, or any history of an allergy to intravenous immunoglobulin. In addition, all participants were rigorously screened to exclude those with prior or current viral exposure, active or latent infections, or any inflammatory condition. Specifically, enrollment required no evidence of viral exposure, including negative screening for hepatitis B virus (HBV), hepatitis C virus (HCV), human immunodeficiency virus (HIV), and tuberculosis (TB), as well as absence of acute or chronic inflammatory diseases. Sexually active men with a partner who could become pregnant and women who could become pregnant in the absence of an effective method of birth control, or who were pregnant or breastfeeding, were also excluded. All participants were required to use two effective forms of contraception, at least one of which had to be a barrier method. Additional details of inclusion and exclusion criteria are outlined in the trial protocol. Participants provided written informed consent before screening.

### STSA-1002 drug product

The STSA-1002 drug product was manufactured at Beijing JOINN Biologics Co., Ltd with a recombinant Chinese hamster ovary cell line.

### Dosing

Cohorts 1-3 received four intravenous infusions (7 days apart) of STSA-1002 at doses of 5 mg/kg (cohort 1), 10 mg/kg (cohort 2), and 20 mg/kg (cohort 3) and were monitored for 56 days. Health volunteers were sequentially recruited into three cohorts and then randomized to receive STSA-1002 or a placebo. There was no difference in appearance between STSA-1002 and placebo. STSA-1002 was administered as an intravenous infusion. Before use, STSA-1002 was reconstituted with saline. Dose infusion volumes were 250 mL, and Placebo (identical to the investigational drug in all components except for the STSA-1002 antibody) was administered as an intravenous infusion in an equivalent volume to the active treatment. All infusions were given over a 2 h period using a peristaltic infusion pump.

### Assessments

#### PK, PD, and ADA assessment

Blood samples were collected for PK, PD, and ADA assessment. The PK samples were collected within 1 h before each of the four doses, at 0 h, 2 h, 6 h, 12 h, 24 h, 48 h, 72 h after the end of the first and the fourth dose, respectively; and additionally at 28 days, 35 days, 42 days, and 56 days. The PD samples were collected within 1 h before each of the four doses, at 2 h, 12 h, 24 h, 72 h after the end of the first and the fourth dose, respectively; and additionally at 28 days, 35 days, 42 days, and 56 days. ADA samples were collected within 1 h before the first dose, D28, and D56.

PK, PD, and ADA were analyzed at JIONN Medical Testing Laboratories (Beijing) Co., Ltd. using validated ELISA methods.

STSA-1002 was administered intravenously, and plasma concentrations reached peak almost immediately after the end of infusion. Therefore, a sample was taken at 0 h post-dose to accurately capture C_max_. To characterize the elimination phase after the first dose, samples were collected within the first 3 days and on Day 7, allowing calculation of initial PK parameters. Based on non-clinical data, the terminal half-life in healthy subjects after multiple doses was expected to increase with dose; for the 20 mg/kg dose, the half-life was projected to exceed 10 days. Consequently, sparse PK sampling was performed up to Day 56 (i.e., >5 half-lives after the last dose), with more frequent sampling during the first 3 days and less frequent sampling thereafter to capture the full time-course while minimizing participant burden.

#### Exploratory Indicators assessment

Exploratory Indicators assessments included measurement of myeloperoxidase (MPO), recombinant neutrophil elastase (NE), proteinase 3 (PR3), and CXC chemokine receptor 1 (CXCR1) in serum using the ELISA method. Samples were collected within 1 h before each of the four doses, at 24 h, 72 h after the first and fourth doses, D28, and D56 post-dose by ELISA. All sample analyses were conducted by JIONN Medical Testing Laboratories (Beijing) Co., Ltd.

#### Safety

Safety was assessed based on the frequency and severity of adverse events (AEs), serious AEs (SAEs), and treatment-emergent adverse events (TEAEs, defined as AEs occurring during the treatment phase). Clinical laboratory tests, electrocardiographic monitoring, vital signs, and physical examinations were evaluated throughout the study period or until the participant had been deemed lost to follow-up. All individuals who were enrolled and received at least 1 dose of the study drug were included in the safety and tolerability analysis. All AEs were followed by the investigator until satisfactory resolution or a clinically stable endpoint. Laboratory abnormalities were determined according to the criteria specified in the Common Terminology Criteria for Adverse Events (CTCAE 5.0) and in accordance with the normal ranges of the clinical laboratory if no grading was available. AEs were assessed regularly throughout the study, were coded according to the Medical Dictionary for Regulatory Activities (MedDRA v25.1), and were assumed to be treatment emergent unless there was clear evidence that the event was present at the first dose of study drug.

#### Statistical analysis

The sample size for this first-in-human study was determined based on empirical considerations rather than formal statistical power calculations. The cohort sizes were chosen to obtain adequate safety and preliminary pharmacokinetic/pharmacodynamic (PK/PD) data to inform the design of future phase I studies with STSA-1002, while minimizing unnecessary exposure of healthy volunteers to the investigational drug. Descriptive statistics were used to summarize continuous variables, including number of observations, mean, geometric mean, standard deviation (SD), coefficient of variation (%CV), median, minimum, and maximum. Categorical variables were summarized using frequency counts and percentages. The safety population included all participants who received any amount of study drug infusion. The PK population comprised a subset of the safety population with evaluable PK profiles, excluding participants with major protocol deviations or other factors that could confound the PK of STSA-1002.

## Results

### Binding affinity and specificity of STSA-1002

As summarized in Table 1, STSA-1002 bound to human C5a with high affinity, exhibiting a binding affinity (KD) of 1.05 × 10^−10^ M, with a Kon of 4.02 × 10^5^ M^-1^s^-1^ and a Koff of 4.22 × 10^−5^ s^-1^. In contrast, no binding signal was detected between STSA-1002 and human C5, indicating a KD greater than 1 × 10^−7^ M. These results demonstrated that STSA-1002 was a high-affinity and highly selective monoclonal antibody targeting human C5a. All measurements were performed in three independent experiments, each including duplicate injections of each concentration. The representative sensorgrams from one experiment are shown. The goodness-of-fit was assessed using the criteria described above.

**TABLE 1:** Characterization of the binding affinity of STSA-1002.

Antibody	Antigen	Kon (M^-1^s^-1^)	Koff (s^-1^)	KD(M)
STSA-1002	C5	No signal	No signal	>1 × 10^−7^
	C5a	4.02 × 10^5^	4.22 × 10^−5^	1.05 × 10^−10^

Abbreviations: Kon: association rate constant; Koff: dissociation rate constant; KD: equilibrium dissociation constant; C5: complement component 5; C5a: complement component 5a.

### Inhibition of C5a-mediated ROS production by STSA-1002

**FIGURE 1:**
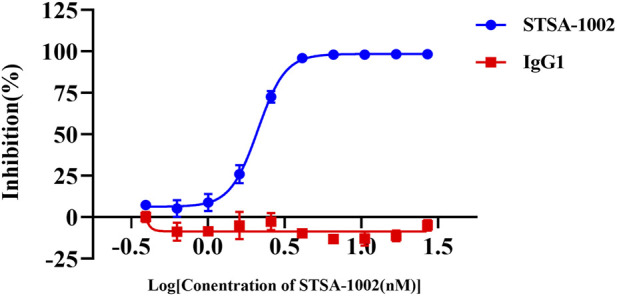
STSA-1002-mediated blockade of ROS production in HL-60 cells (n = 3). Abbreviations: ROS: reactive oxygen species; HL-60: human promyelocytic leukemia cells.

The ability of STSA-1002 to block C5a-mediated production of reactive oxygen species (ROS) in HL-60 cells was evaluated by using the cell-permeant 2′7′-dichloroflurescin diacetate (DCFDA) quantitative fluorometric method. The results demonstrated that an average IC50 was 2.11 nM in the STSA-1002 group, whereas the isotype control (IgG1)did not affect ROS production in HL-60 cells. These data demonstrated that STSA-1002 potently blocked C5a-mediated production of ROS in HL-60 cells. STSA-1002 can effectively block C5a stimulation of neutrophils to release reactive oxygen species (ROS). The data were normalized to calculate the inhibitory activity of the antibody, with only C5a stimulation and free of C5a stimulation as 0% and 100% inhibition, respectively. The experiment was performed three times (n = 3). Inhibition curves were fitted using GraphPad Prism software with a four-parameter logistic (log (inhibitor) vs. response -Variable Slope) model to calculate the IC_50_. The mean IC_50_ value was then calculated from the three independent experiments. The ROS production inhibitory activity of the antibodies was shown in Figure 1.

### Patient disposition

**FIGURE 2:**
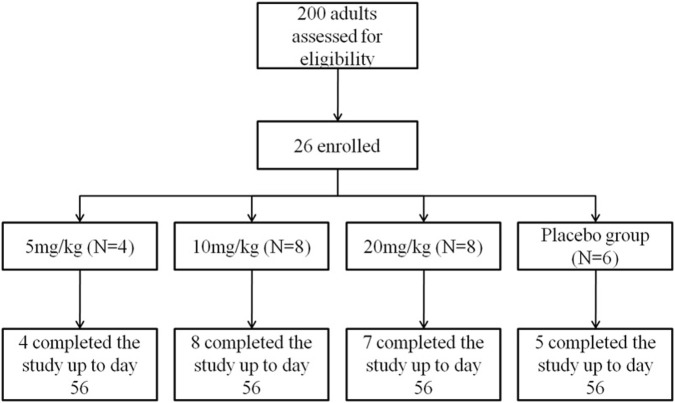
Patient disposition.

The study was conducted from October 2022 until April 2023. Of 200 patients enrolled, 26 were randomized and 26 received treatment (n = 20, STSA-1002; n = 6, placebo). Six subjects were randomized (STSA-1002: placebo = 2:1) in the 5 mg/kg cohort, whereas 10 subjects per cohort were randomized (4:1) in the 10 mg/kg and 20 mg/kg cohorts.1 patient dropped out of the trial due to personal reasons after receiving the drug in the first and second period, and was excluded from PK analyses. Overall, 25 patients (96.2%) completed the treatment period, and 24 (92.3%) completed the 56-day study period (Figure 2). The mean age ranged from 31.1 to 37.0 years, and mean BMI ranged from 22.58 to 23.65 kg/m^2^ in the intention-to-treat population. Baseline characteristics of the randomly assigned participants were shown in Table 2.

**TABLE 2:** Baseline characteristics of the intention-to-treat population.

Demographic characteristics	STSA-1002 dose			Total STSA-1002 (N = 20)	Placebo (N = 6)
	5 mg/kg (N = 4)	10 mg/kg (N = 8)	20 mg/kg (N = 8)		
Age (years)					
Mean (SD)	37.0 (5.0)	36.5 (3.1)	31.1 (7.5)	34.5 (6.0)	29.3 (5.0)
Median	37.5 (33.0, 41.0)	36.0 (34.0, 38.5)	28.5 (26.0, 38.0)	35.0 (31.0, 39.5)	30.5 (25.0, 33.0)
Sex (n %)					
Female	2 (50.00)	2 (25.00)	3 (37.50)	7 (35.00)	1 (16.67)
Male	2 (50.00)	6 (75.00)	5 (62.50)	13 (65.00)	5 (83.33)
Weight (kg)					
Mean (SD)	65.48 (7.91)	61.19 (6.15)	65.29 (6.13)	63.69 (6.48)	66.22 (7.07)
Median	65.10 (59.25, 71.70)	61.80 (55.55, 65.75)	65.85 (61.80, 68.70)	63.00 (59.00, 68.50)	67.95 (59.40, 71.50)
Weight at baseline ^[1]^ (kg)					
Mean (SD)	65.18 (7.21)	61.29 (6.16)	64.24 (6.36)	63.25 (6.31)	65.28 (7.14)
Median	65.30 (59.80, 70.55)	61.65 (56.00, 65.95)	64.25 (60.10, 68.20)	62.60 (58.55, 67.90)	66.60 (58.20, 71.30)
BMI (kg/m^2^)					
Mean (SD)	23.65 (1.00)	22.58 (2.05)	23.65 (1.70)	23.22 (1.75)	22.90 (1.72)
Median	23.35 (23.05, 24.25)	22.50 (20.85, 24.50)	23.50 (22.65, 25.05)	23.35 (21.80, 24.50)	23.25 (21.80, 24.10)

[1] Weight at baseline: Weight at baseline is defined as the last non-null test result before the first dose of the drug.

Abbreviations: SD: standard deviation. BMI: body-mass index.

The sample size and allocation ratio varied between dose groups based on safety considerations and the estimated effective dose range. The low dose group (5 mg/kg) was designed as a transition from single to multiple dose administration, where the effects of repeated dosing in humans were not yet fully known; therefore, a smaller cohort with a 2:1 randomization (active: placebo) was adopted to minimize risk to participants, and dose escalation proceeded only after no serious adverse events were observed. In contrast, the medium and high dose groups (10 and 20 mg/kg) fell within the predicted effective dose range, warranting a larger number of subjects on active treatment, and thus a 4:1 randomization ratio was used to obtain more robust safety and PK/PD data for these clinically relevant dose levels.

### Safety

#### Adverse events

Across all cohorts, a total of 24 (92%) participants reported at least one TEAE during the study, with 2 (50.0%), 8 (100.0%), 8 (100.0%) and 6 (100%) participants experiencing TEAEs in the 5-, 10-, 20-mg/kg, and placebo cohorts (2 placebo participants in each dose cohort), respectively. There was one grade 3 adverse event of neutrophil count in the placebo cohort, and other AEs were all grade 1–2.

The most common adverse events were blood triglycerides increased (n = 5 [25%] in participants who received STSA-1002 and n = 1 [16.7%] in those receiving placebo), neutrophil count decreased (n = 3 [15.0%] vs. n = 2 [33.3%]), alanine aminotransferase increased (n = 2 [10.0%] vs. n = 0), urine ketone body present (n = 2 [10.0%] vs. n = 0), haemoglobin decreased (n = 2 [10.0%] vs. n = 0) diarrhoea (n = 2 [10.0%] vs. n = 0), COVID-19 (n = 8 [40%] vs. n = 2 [33.3%]) and Influenza (n = 5 [25%] vs. n = 2 [33.3%]) (Table 3). Due to the epidemics of COVID-19 and influenza during the trial in China, most subjects in both the STSA-1002 and placebo groups contracted mild to moderate infections, which were assessed as unrelated to the investigational product. All COVID-19 and influenza events were reported in the 10 mg/kg and 20 mg/kg cohorts (including the investigator product or placebo participants), while no infectious events were reported in the 5 mg/kg cohort. This may cause certain interference with the evaluation of safety data in trial participants.

**TABLE 3:** Number of TEAEs by Grade and Common AEs (≥10% in total STSA-1002 group).

AEs	STSA-1002 dose			Total STSA-1002 (N = 20)	Placebo (N = 6)
	5 mg/kg (N = 4)	10 mg/kg (N = 8)	20 mg/kg (N = 8)		
TEAE (SOC/PT)	2 (50.0%)	8 (100.0%)	8 (100.0%)	18 (90.0%)	6 (100.0%)
AE with grade 1	1 (25.0%)	1 (12.5%)	3 (37.50)	5 (25.00)	2 (33.33)
AE with grade 2	1 (25.0%)	7 (87.5%)	5 (62.5%)	13 (65.0%)	3 (50.0%)
TRAE with grade 2	1 (25.0%)	1 (12.5%)	2 (25.0%)	4 (20.0%)	2 (33.3%)
AE with grade≥3	0.00	0.00	0.00	0.00	1 (16.7%)
Common AEs (≥10% in total STSA-1002 group) (PT)					
Blood triglycerides increased	1 (25.0%)	2 (25.0%)	2 (25.0%)	5 (25.0%)	1 (16.7%)
Neutrophil count decreased	1 (25.0%)	0.00	2 (25.0%)	3 (15.0%)	2 (33.3%)
Alanine aminotransferase increased	0.00	1 (12.5%)	1 (12.5%)	2 (10.0%)	0.00
Urine ketone body present	0.00	1 (12.5%)	1 (12.5%)	2 (10.0%)	0.00
Haemoglobin decreased	0.00	1 (12.5%)	1 (12.5%)	2 (10.0%)	0.00
COVID-19	0.00	8 (100.0%)	0.00	8 (40.0%)	2 (33.3%)
Influenza	0.00	0.00	5 (62.5%)	5 (25.0%)	2 (33.3%)
Diarrhoea	0.00	0.00	2 (25.0%)	2 (10.0%)	0.00

Data are the number of participants (%).

Abbreviations: TEAE: treatment-emergent adverse event; SOC: system organ class; PT: preferred term; AE: adverse event; QT: QT, interval; COVID-19: coronavirus disease 2019.

Most of the AEs were mild and transient during the study period. No serious adverse event (SAE) or AE leading to study withdrawal or dose escalation discontinuation was observed.

#### Pharmacokinetics of STSA-1002

Serum concentration analysis was performed in subjects who received STSA-1002 injection and had at least one valid post-dose concentration measurement. PK parameter analysis was conducted in subjects who received STSA-1002 injection, had at least one valid PK parameter, and did not experience any protocol deviations that significantly impacted PK evaluation. 20 subjects who received at least one dose were included for serum concentration and PK parameter analysis. 19 subjects who completed all four doses were included for serum concentration and PK parameter analysis.

Pharmacokinetic analysis showed that the mean (±SD) C_max_ after the first dose (C_max, D0_) were 137.67 ± 19.57, 279.11 ± 25.88, and 445.13 ± 33.0 μg/mL for 5 mg/kg, 10 mg/kg, and 20 mg/kg, respectively (Table 4).

**TABLE 4:** Pharmacokinetic parameters for the first administration (Mean ± SD).

Groups	T_1/2_ (h)	C_max_ (μg/mL)	AUC_last_ (h × μg/mL)	${\text{AUC}}_{0-\infty }$ (h × μg/mL)	V_z_ (mL)	Clearance (mL/h)
5 mg/kg (n = 4)	50.18 ± 9.21	137.67 ± 19.57	10257.46 ± 366.85	11339.21 ± 315.96	2151.62 ± 265.87	30.03 ± 2.96
10 mg/kg (n = 4)	NA (NA)	279.11 ± 25.88	23596.75 ± 2982.71	NA (NA)	NA (NA)	NA (NA)
20 mg/kg (n = 8)	NA (NA)	445.13 ± 33.00	41173.60 ± 2397.58	NA (NA)	NA (NA)	NA (NA)

Abbreviations: SD: standard deviation; T_1/2_: half-life; C_max_: maximum concentration; AUC_last_: area under the concentration time-curve from time of dose to last sample time point; ${\text{AUC}}_{0-\infty }$ : area under the concentration time-curve from 0 h to infinity; Vz: volume of distribution during the terminal phase.

**FIGURE 3:**
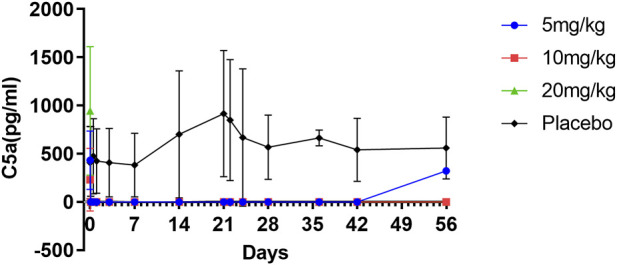
The mean concentration-time profile of STSA-1002 in the 5 mg/kg, 10 mg/kg, and 20 mg/kg cohorts (semi-logarithmic) (n = 20). Healthy subjects received STSA-1002 at 5 mg/kg (n = 4), 10 mg/kg (n = 8), or 20 mg/kg (n = 8) once weekly for 4 weeks. Blood samples were collected pre-dose and at multiple time points up to Day 56. Plasma concentrations of STSA-1002 were measured using a validated ELISA method. Data are presented as mean ± standard deviation (SD), with error bars representing SD. The y-axis is shown on a logarithmic scale (base 10) to accommodate the wide concentration range. No statistical comparisons were performed for this figure; the plot is intended for descriptive illustration of the pharmacokinetic profiles. The y-axis is shown on a logarithmic scale (base 10).

After the fourth dose, the mean (±SD) C_max, D21_ were 209.34 ± 15.04, 381.86 ± 75.03, and 908.66 ± 131.01 μg/mL for 5 mg/kg, 10 mg/kg, and 20 mg/kg, respectively (Table 5). The mean (±SD) AUC_0-t, D21_ after four IV infusions were 34930.72 ± 1236.22, 106151.19 ± 44094.55, and 333207.26 ± 42802.34 h*μg/mL for 5 mg/kg, 10 mg/kg, and 20 mg/kg, respectively. The mean (±SD) ${\text{AUC}}_{0-\infty }$ _, D21_ after four IV infusions were 35789.07 ± 1909.31, 100167.19 ± 45728.59, and 360812.95 ± 45806.00 h*μg/mL for 5 mg/kg, 10 mg/kg, and 20 mg/kg, respectively. The t_1/2_ of STSA-1002 was 4.26 ± 0.61 days for 5 mg/kg, 5.41 ± 3.83 days for 10 mg/kg, and 12.08 ± 0.89days for 20 mg/kg. After the last administration, AUC_0-D7, D21,_ and ${\text{AUC}}_{0-\infty ,D21}$ increased with the dose, and overproportionally increased with dosage. Accumulation factor was 1.38–2.00 and 1.80–2.70 calculated with C_max_ and AUC, respectively, indicating a mild accumulation after multiple intravenous administrations in each dose group. The mean concentration *versus* time profile of STSA-1002 is shown in Figure 3.

**TABLE 5:** Pharmacokinetic parameters for the last administration (Mean ± SD).

Groups	T_1/2_ (days)	C_max_ (μg/mL)	AUC_last_ (h × μg/mL)	${\text{AUC}}_{0-\infty }$ (h × μg/mL)	V_z_ (mL)	Clearance (mL/h)
5 mg/kg (n = 4)	4.26 ± 0.61	209.34 ± 15.04	34930.72 ± 1236.22	35789.07 ± 1909.31	1331.77 ± 158.82	9.12 ± 1.15
10 mg/kg (n = 4)	5.41 ± 3.83	381.86 ± 75.03	106151.19 ± 44094.55	100167.19 ± 45728.59	1084.13 ± 297.41	6.78 ± 1.75
20 mg/kg (n = 8)	12.08 ± 0.89	908.66 ± 131.01	333207.26 ± 42802.34	360812.95 ± 45806.00	1550.81 ± 272.16	3.70 ± 0.52

Abbreviations: SD: standard deviation; T_1/2_: half-life; C_max_: maximum concentration; AUC_last_: area under the concentration time-curve from time of dose to last sample time point; ${\text{AUC}}_{0-\infty }$ : area under the concentration time-curve from 0 h to infinity; V_z_: volume of distribution during the terminal phase; NA: not available.

Power Model was used to analyze the proportionality between AUC_0-t, D0_, AUC_0-t, D21_ and the dose. Both AUC_0-t, D0_, AUC_0-t, D21,_ and dose were log-transformed, and the resulting logarithm values were standardized. Taking AUC_0-t, D0_ and AUC_0-t, D21_ as the dependent variable and dose as the independent variable, the estimated value and its 90% confidence interval (CI), calculated by the model, were 0.97 (0.89, 1.06) and 1.66 (1.42, 1.91) for AUC_0-t, D0_ and AUC_0-t, D21_, respectively (Table 6). Power model analyses demonstrated that the pharmacokinetics of the drug are approximately dose-proportional following the first administration and non-linear following the last administration at the dose range of 5∼20 mg/kg.

**TABLE 6:** Linear Relationship between ln (Dose) and PK Parameters after First Dose.

Parameters (Unit)	Independent variable	Point estimate	Standard error	90% CI
AUC_0-t,D0_ (h*ng/ml)	Dose	0.97	0.05	(0.89,1.06)
	Intercept	14.65	0.12	(14.45,14.86)
${\text{AUC}}_{0-\infty ,D21}$ (h*ng/ml)	Dose	1.66	0.14	(1.42,1.91)
	Intercept	14.63	0.33	(14.05,15.21)

Abbreviations: PK: pharmacokinetic; AUC_0-t,D0_: Area under the concentration-time curve from time zero to the last measurable time point after the first dose; ${\text{AUC}}_{0-\infty ,D21}$ : Area under the concentration-time curve from time zero extrapolated to infinity after the last dose on Day 21; CI: confidence interval.

#### Pharmacodynamics of STSA-1002

**FIGURE 4:**
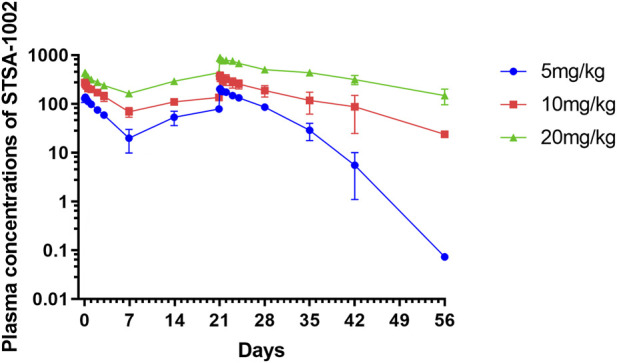
The mean plasma free C5a concentration (±SD)-time curve in the 5 mg/kg, 10 mg/kg and 20 mg/kg STSA-1002 cohorts (n = 26). Healthy subjects received STSA-1002 at 5 mg/kg (n = 4), 10 mg/kg (n = 8), 20 mg/kg (n = 8), or a matching placebo (n = 6) once weekly for 4 weeks. Blood samples were collected pre-dose and at the indicated time points up to Day 56. Plasma C5a concentrations were measured using a validated immunoassay. Data are presented as mean ± standard deviation (SD); error bars represent SD. The y-axis shows C5a concentration in pg/mL on a linear scale. No statistical comparisons are shown in the figure; the plot is intended to illustrate the pharmacodynamic suppression of C5a across treatment groups. Abbreviations: SD: standard deviation.

The mean concentrations of mean (±SD) C5a in baseline were 432.61 ± 302.38, 230.35 ± 325.60, 945.23 ± 667.17, and 407.30 ± 375.96 pg/mL for 5 mg/kg, 10 mg/kg, and 20 mg/kg, respectively. The C5a level decreased rapidly to below the quantitation limit (250 pg/mL) from the first detection point in all treatment groups (Figure 4). In the 5 mg/kg group, the mean free C5a level fell below the quantitation limit from 2 h after the end of the first infusion through Day 42. In the 10 mg/kg and 20 mg/kg groups, concentrations remained below the quantitation limit from 2 h after the end of the first dose until Day 56. In contrast, free C5a was detectable at all time points in the placebo group, with no apparent decrease from baseline levels.

Of note, no appreciable inter-individual variability in free C5a suppression was observed: all subjects in the STSA-1002 treatment groups achieved and maintained levels below the lower limit of quantification (LLOQ) throughout the suppression period (until at least Day 42 for the 5 mg/kg group and Day 56 for the 10 and 20 mg/kg groups). Therefore, error bars at these time points reflect only assay-related minimal variation and not true biological differences.

#### ADA

The ADA samples were collected at pre-dose, D28, and D56. A validated ELISA method was used for ADA detection. The screening sensitivity and confirmatory sensitivity of this method are 19.240 ng/mL and 44.213 ng/mL, respectively. The drug tolerance levels are as follows: When the positive antibody concentration is 2000 ng/mL, the resistance limit is 1000.000 μg/mL. When the positive antibody concentration is 100 ng/mL, the resistance limit is 500.000 μg/mL. Based on all 26 subjects enrolled, no ADA antibodies were detected through Day 56 post-first dose.

#### Serum concentrations of MPO, NE, and PR3

**FIGURE 5:**
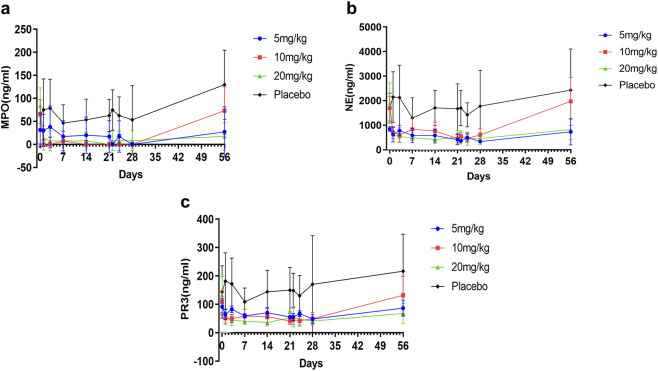
Mean serum concentrations (±SD)-time curve of MPO**(a)**, NE**(b)** and PR3 **(c)**in the 5 mg/kg, 10 mg/kg and 20 mg/kg STSA-1002 cohorts (n = 26). Subjects received STSA-1002 at 5 mg/kg (n = 4), 10 mg/kg (n = 8), or 20 mg/kg (n = 8), or placebo (n = 6), once weekly for 4 weeks. MPO, NE and PR3 levels were measured at the indicated time points up to Day 56. Data are presented as mean ± standard deviation (SD) with error bars representing SD. Abbreviations: SD: standard deviation; MPO: myeloperoxidase; NE: neutrophil elastase; PR3: proteinase 3.

The MPO level ranged from 31.19 ± 36.49 ng/mL to 83.92 ± 38.92 ng/mL before administration. After first-dose, mean MPO serum concentrations in all treatment groups rapidly decreased especially in 10 mg/kg and 20 mg/kg (from baseline to 24 h after the end of dose: 31.19 to 30.30 ng/mL in 5 mg/kg group; 65.72 ng/mL to 0 ng/mL in 10 mg/kg group; and 83.92 ng/mL to 14.36 ng/mL in 20 mg/kg group). The duration of MPO reduction showed a dose-dependent trend: the 5 mg/kg and 10 mg/kg groups returned to baseline levels by Day 56 post-dose, while the 20 mg/kg group gradually recovered but did not reach baseline by Day 56. These results suggested a trend of rapid reduction in serum MPO levels with STSA-1002 (Figure 5).

The baseline level of NE ranged from 843.80 ± 91.32 ng/mL to 1903.01 ± 830.44 ng/mL. NE levels in all treatment groups exhibited a rapid decline, with a dose-dependent trend in the duration of reduction. After the first dose, mean NE serum concentrations quickly decreased to almost 30% of the baseline level in all treatment groups (617.00 ng/mL ∼ 709.21 ng/mL). The 5 mg/kg and 10 mg/kg groups almost recovered to baseline levels by Day 56 post-administration, while the 20 mg/kg group showed gradual recovery by Day 56 but did not return to baseline levels. The data were suggestive of a reduction trend where NE levels differed between the STSA-1002 cohorts and the placebo group (Figure 5).

The baseline serum PR3 level ranged from 90.99 ± 28.82 ng/mL to 132.89 ± 64.56 ng/mL across treatment groups. Following the first dose, PR3 levels rapidly decreased to approximately 30% of baseline in all treatment groups. The subsequent time course of suppression exhibited a clear dose-dependent trend. While PR3 levels in the 5 mg/kg and 10 mg/kg groups fluctuated within the suppressed range through Day 28 and recovered to near or above baseline by Day 56, the 20 mg/kg group demonstrated a prolonged inhibitory effect, with levels remaining suppressed through Day 28 and failing to return to baseline by Day 56. In contrast, PR3 concentrations in the placebo group increased from baseline over the same period. These data suggested a differential effect of STSA-1002 on PR3 levels compared to placebo, characterized by a dose-dependent duration of suppression (Figure 5).

## Discussion

The primary objective of this Phase I study was to assess the safety and tolerability of STSA-1002 following multiple intravenous administrations in healthy volunteers. All 26 randomized subjects completed the trial per protocol. STSA-1002 was found to be safe and well-tolerated at doses up to 20 mg/kg administered once weekly for four consecutive weeks. Notably, the overall frequency and pattern of treatment-emergent adverse events were similar between active treatment and placebo groups, with no safety signals identified that would preclude further clinical development.

STSA-1002 demonstrated a favorable safety and tolerability profile in healthy adult participants following four weekly intravenous doses at 5, 10, and 20 mg/kg. Most treatment-emergent adverse events (TEAEs) were mild in intensity. The most frequently TEAEs included laboratory abnormalities (elevated blood triglycerides, decreased neutrophil count, increased alanine aminotransferase, and presence of urine ketone bodies) as well as diarrhea. No clinically significant abnormalities in electrocardiograms or vital signs were observed, and there were no safety signals that would preclude further clinical development.

The selection of doses based on the preclinical data for this first-in-human, multiple-dose study was based on multiple factors, including data from human primate models and non-clinical pharmacokinetic and safety data. Before clinical studies, GLP-compliant, repeat-dose, toxicology studies were done in rhesus macaques at Joinn Biologics (Beijing, China).

In a 4-week repeat-dose toxicity study, naïve animals received intravenous infusions of STSA-1002 at 0, 20, 60, or 200 mg/kg. The dosing schedule comprised five administrations during the first week, followed by three weekly doses for three consecutive weeks (15 doses total). STSA-1002 was well tolerated across all dose levels, with the no-observed-adverse-effect level (NOAEL) established as≥200 mg/kg.

In a separate 2-week repeat-dose toxicity study, animals received a total of eight doses (five in week 1 and three in week 2) at 30, 100, or 300 mg/kg. The maximum tolerated dose (MTD) was determined to be 300 mg/kg. Drug accumulation was observed in all dose groups, with mean accumulation ratios (AUC_0-24, Day 13_/AUC_0-24, Day 1_) of 4.21, 3.82, and 3.20, respectively. Based on the collective preclinical findings demonstrating a wide safety margin, a maximum clinical dose of 20 mg/kg was selected for evaluation in this first-in-human trial.

STSA-1002 was initially evaluated in a Phase Ia single-ascending-dose (SAD) study in healthy subjects (NCT05032144). Results demonstrated a favorable safety and tolerability profile across the dose range of 2–30 mg/kg. Most treatment-emergent adverse events were mild to moderate in severity (Grade 1 or 2), with no drug-related serious adverse events (SAEs) reported. Importantly, no adverse events necessitated participant withdrawal or interruption of dose escalation. Even at the highest tested dose of 30 mg/kg, STSA-1002 maintained an acceptable safety profile. Pharmacokinetic analysis showed that plasma concentrations increased in an approximately dose-proportional manner following single-dose administration across the 2 mg/kg - 30 mg/kg range. These Phase Ia findings informed the design of the subsequent Phase Ib multiple-dose study, with a starting dose of 5 mg/kg and a maximum dose of 20 mg/kg selected for further evaluation. Dose escalation was conducted in accordance with predefined stopping rules, proceeding to the next cohort only after all subjects in each dose group had completed the Day 14 safety assessment following their last administration.

STSA-1002 presents several potential advantages for the treatment of v-ARDS. First, it exhibits potent and sustained inhibition of its target, C5a. A single administration, particularly at doses of 10 and 20 mg/kg, achieves robust C5a suppression, with an inhibition duration that is both dose-dependent and extended upon repeated dosing. Second, STSA-1002 can specifically bind to C5a, causing C5a to lose its ability to bind to the receptor (C5aR1). This results in effective inhibition of C5a- and C5a-desArg-induced CD11b upregulation on human neutrophils (Yingying Fang and Wang, 2024; Fang et al., 2025) and, in a concentration-dependent manner, suppresses the production of reactive oxygen species (ROS) - a key mediator of ARDS-related lung injury (Spadaro et al., 2019; Lim et al., 2023). Finally, the translational potential of STSA-1002 is supported by *in vivo* efficacy data from a COVID-19-associated ARDS mouse model, where a single injection at 1, 3, or 10 mg/kg significantly reduced mortality. Interestingly, there were extremely different changes of MPO, NE, and PR3 between the STSA-1002 groups and the placebo group, which was different from the recent study. MPO, NE, and PR3 were founded to be special biomarkers of NETs. As part of NETs released by activated neutrophils, MPO, NE, and PR3 decorate a meshwork of decondensed chromatin fibers, enabling NETs to entrap and neutralize pathogens (Pisareva et al., 2022; Islam and Takeyama, 2023). However, in the exploratory substudy of the phase 2 PANAMO study, the blood protein plasma markers of NET formation did not differ significantly between the anti-C5a antibody vilobeliab and the placebo group (Lim et al., 2022). The discrepancy between our observations and the phase 2PANAMO study may be attributed to the distinct biomarkers assessed. The PANAMO substudy focused on tissue factor (TF) to evaluate the role of TF-bearing NETs in thrombotic activity (Skendros et al., 2020; de Bruin et al., 2021), whereas our analysis measured the core NET structural components (MPO, NE, PR3). Furthermore, methodological differences may contribute, as the present study evaluated a fixed regimen of four STSA-1002 infusions at set intervals, whereas treatment schedules in the PANAMO trial involved variable dosing intervals over 5-7 administrations. Critically, in healthy individuals, C5a inhibition may produce a clearer relative change in NET biomarkers from a low baseline, whereas in critically ill patients, persistent activation of multiple inflammatory pathways may overwhelm or compensate for C5a blockade, potentially explaining the lack of effect of vilobelimab in the PANAMO substudy.

There are several potential limitations of the STSA-1002 trial. First, as a Phase I trial, the study enrolled a relatively small number of participants, limiting the generalizability of the findings and precluding definitive conclusions regarding safety in broader populations. Nonetheless, the consistency of these results with prior Phase I studies of STSA-1002 lends confidence to the observed safety and pharmacokinetic profiles. Second, the follow-up period was limited to 56 days after the last dose, which may not capture very late-onset adverse events or the full duration of pharmacodynamic effects beyond this window. Notably, this study was designed to evaluate safety, tolerability, pharmacokinetics, and pharmacodynamics, and was not powered to assess clinical efficacy. Third, the observed non-linear pharmacokinetics, characterized by a decrease in clearance and prolongation of half-life with increasing doses, are consistent with target-mediated drug disposition (TMDD), a common feature of monoclonal antibodies targeting soluble ligands such as C5a. While this TMDD-related non-linearity enhances target suppression and may support less frequent dosing, it also necessitates careful dose selection in future Phase II trials to avoid unexpected drug accumulation and to define the optimal therapeutic window. In addition, during the trial period, there were mild COVID-19 and influenza infections in some participants. Although these infections were balanced between the STSA-1002 and placebo groups and were not considered drug-related, their occurrence could potentially confound the safety assessment to a limited extent. Given the small sample size and the exploratory nature of this Phase I trial, a formal sensitivity analysis or subgroup analysis by infection status was not feasible due to the very small number of infected subjects in each group. Therefore, the impact of intercurrent viral infections on the safety profile could not be fully isolated. Future Phase II/III trials with larger sample sizes will be better suited to evaluate this potential confounder systematically. Moreover, in the 20 mg/kg group, three subjects had plasma concentrations slightly above the drug tolerance limit at Day 28 (518–582 μg/mL), which may have compromised ADA detection at that single time point. Nevertheless, all baseline and Day 56 samples were within the tolerance limit, and no ADA positivity was detected at any time point, supporting the overall conclusion of no immunogenicity. Finally, the pharmacodynamic responses (including suppression of C5a and modulation of NET-associated biomarkers) were derived from a small Phase I study in healthy volunteers; therefore, definitive conclusions regarding the drug’s effect on C5a and NETosis in viral ARDS cannot be drawn. Validation in patient populations with active inflammation is required in future studies. Therefore, while the pharmacodynamic effects observed are promising, the therapeutic potential of STSA-1002 in v-ARDS awaits confirmation in appropriately designed Phase IIb efficacy trials.

## Conclusion

STSA-1002 was generally safe and well-tolerated, with no observed immunogenicity when administered in multiple intravenous doses. These findings support the continued development of STSA-1002 for the treatment of v-ARDS.

## Data Availability

The original contributions presented in the study are included in the article/supplementary material, further inquiries can be directed to the corresponding author.

## References

[B1] Carvelli J. Demaria O. Vély F. Batista L. Chouaki Benmansour N. Fares J. (2020). Association of COVID-19 inflammation with activation of the C5a-C5aR1 axis. Nature 588, 146–150. 10.1038/s41586-020-2600-6 32726800 PMC7116884

[B2] de Bruin S. Bos L. D. van Roon M. A. Tuip-de Boer A. M. Schuurman A. R. Koel-Simmelinck M. (2021). Clinical features and prognostic factors in Covid-19: a prospective cohort study. EBioMedicine 67, 103378. 10.1016/j.ebiom.2021.103378 34000622 PMC8118723

[B3] Fang Y. Wang X. Xu C. Zhu Q. Zang X. Liu J. (2025). Preclinical evaluation of STSA-1002, a novel human and rhesus monkeys cross-reactive monoclonal antibody targeting C5a, in acute respiratory distress syndrome models. Int. Immunopharmacol. 164, 115338. 10.1016/j.intimp.2025.115338 40848483

[B4] Garcia C. C. Weston-Davies W. Russo R. C. Tavares L. P. Rachid M. A. Alves-Filho J. C. (2013). Complement C5 activation during influenza A infection in mice contributes to neutrophil recruitment and lung injury. PLoS. ONE 8, e64443. 10.1371/journal.pone.0064443 23696894 PMC3655967

[B5] Grotberg J. C. Reynolds D. Kraft B. D. (2023). Management of severe acute respiratory distress syndrome: a primer. Crit. Care London, Engl. 27, 289. 10.1186/s13054-023-04572-w 37464381 PMC10353255

[B6] Islam M. M. Takeyama N. (2023). Role of neutrophil extracellular traps in health and disease pathophysiology: recent insights and advances. Int. J. Mol. Sci. 24, 15805. 10.3390/ijms242115805 37958788 PMC10649138

[B7] Jiang Y. Li J. Teng Y. Sun H. Tian G. He L. (2019). Complement receptor C5aR1 inhibition reduces pyroptosis in hDPP4-Transgenic mice infected with MERS-CoV. Viruses 11, 39. 10.3390/v11010039 30634407 PMC6356766

[B8] Lim E. Vlaar A. Bos L. van Vught L. A. Boer A. Dujardin R. (2022). Anti-C5a antibody vilobelimab treatment and the effect on biomarkers of inflammation and coagulation in patients with severe COVID-19: a substudy of the phase 2 PANAMO trial. Respir. Res. 23, 375. 10.1186/s12931-022-02278-1 36566174 PMC9789513

[B9] Lim E. Y. Lee S. Y. Shin H. S. Kim G. D. (2023). Reactive oxygen species and strategies for antioxidant intervention in acute respiratory distress syndrome. Antioxidants Basel, Switz. 12, 2016. 10.3390/antiox12112016 38001869 PMC10669909

[B10] Meliopoulos V. A. Karlsson E. A. Kercher L. Cline T. Freiden P. Duan S. (2014). Human H7N9 and H5N1 influenza viruses differ in induction of cytokines and tissue tropism. J. Virol. 88, 12982–12991. 10.1128/JVI.01571-14 25210188 PMC4249090

[B11] Middleton E. A. He X. Y. Denorme F. Campbell R. A. Ng D. Salvatore S. P. (2020). Neutrophil extracellular traps contribute to immunothrombosis in COVID-19 acute respiratory distress syndrome. Blood 136, 1169–1179. 10.1182/blood.2020007008 32597954 PMC7472714

[B12] Pisareva E. Mihalovičová L. Pastor B. Kudriavtsev A. Mirandola A. Mazard T. (2022). Neutrophil extracellular traps have auto-catabolic activity and produce mononucleosome-associated circulating DNA. Genome Med. 14, 135. 10.1186/s13073-022-01125-8 36443816 PMC9702877

[B13] Skendros P. Mitsios A. Chrysanthopoulou A. Mastellos D. C. Metallidis S. Rafailidis P. (2020). Complement and tissue factor-enriched neutrophil extracellular traps are key drivers in COVID-19 immunothrombosis. J. Clin. Invest. 130, 6151–6157. 10.1172/JCI141374 32759504 PMC7598040

[B14] Song N. Li P. Jiang Y. Sun H. Cui J. Zhao G. (2018). C5a receptor1 inhibition alleviates influenza virus-induced acute lung injury. Int. Immunopharmacol. 59, 12–20. 10.1016/j.intimp.2018.03.029 29621732

[B15] Spadaro S. Park M. Turrini C. Tunstall T. Thwaites R. Mauri T. (2019). Biomarkers for acute respiratory distress syndrome and prospects for personalised medicine. J. Inflammation Lond. Engl. 16, 1. 10.1186/s12950-018-0202-y 30675131 PMC6332898

[B16] van der Velden S. van Osch T. Seghier A. Bentlage A. Mok J. Y. Geerdes D. M. (2024). Complement activation drives antibody-mediated transfusion-related acute lung injury *via* macrophage trafficking and formation of NETs. Blood 143, 79–91. 10.1182/blood.2023020484 37801721

[B17] Vlaar A. Witzenrath M. van Paassen P. Heunks L. Mourvillier B. de Bruin S. (2022). Anti-C5a antibody (vilobelimab) therapy for critically ill, invasively mechanically ventilated patients with COVID-19 (PANAMO): a multicentre, double-blind, randomised, placebo-controlled, phase 3 trial. Lancet Respir. Med. 10, 1137–1146. 10.1016/S2213-2600(22)00297-1 36087611 PMC9451499

[B18] Wang R. Xiao H. Guo R. Li Y. Shen B. (2015). The role of C5a in acute lung injury induced by highly pathogenic viral infections. Emerg. Microbes Infect. 4, e28. 10.1038/emi.2015.28 26060601 PMC4451266

[B19] Yingying Fang X. W. Wang X. (2024). STSA1002, a Novel Human and Rhesus Monkeys cross-reactive C5a Monoclonal Antibody for an Immunomodulatory Therapy.

[B20] Zhang Y. Han K. Du C. Li R. Liu J. Zeng H. (2021). Carboxypeptidase B blocks *ex vivo* activation of the anaphylatoxin-neutrophil extracellular trap axis in neutrophils from COVID-19 patients. Crit. Care London, Engl. 25, 51. 10.1186/s13054-021-03482-z 33557911 PMC7868871

